# SNG100, a novel topical treatment for moderate atopic dermatitis, in patients aged 6 years or older: A randomised, double‐blind, active‐controlled trial

**DOI:** 10.1002/ski2.293

**Published:** 2023-10-14

**Authors:** Liat Samuelov, Avner Shemer, Shoshana Greenberger, Inbal Ziv, Doron Friedman, Oron Yacoby‐Zeevi, Roni Dodiuk‐Gad, Yuval Ramot, Sari Murad, Eli Sprecher

**Affiliations:** ^1^ Division of Dermatology Tel Aviv Sourasky Medical Center Tel Aviv Israel; ^2^ Faculty of Medicine Tel Aviv University Tel Aviv Israel; ^3^ Department of Dermatology Sheba Medical Center Ramat‐Gan Israel; ^4^ Department of Dermatology Pediatric Dermatology Unit Sheba Medical Center Ramat Gan Israel; ^5^ Seanergy Dermatology Rehovot Israel; ^6^ Dermatology and Venereology Department Emek Medical Center Afula Israel; ^7^ Ruth and Bruce Rappaport Faculty of Medicine Technion Institute of Technology Haifa Israel; ^8^ Division of Dermatology Department of Medicine University of Toronto Toronto Ontario Canada; ^9^ Department of Dermatology Hadassah‐Hebrew University Medical Center Jerusalem Israel; ^10^ Faculty of Medicine Hebrew University of Jerusalem Jerusalem Israel; ^11^ Dermatology Unit Kaplan Medical Center Rehovot Israel

## Abstract

**Background:**

Atopic dermatitis (AD) is one of the most common inflammatory skin diseases. It is associated with significant itch and impaired quality of life. Systemic treatments are efficient but associated with side effects. Novel topical treatments with a favourable safety profile are needed. SNG100 is a novel composition of hydrocortisone 1% in a cream base comprising sulphated polysaccharide (SPS; extracted from in‐house cultivated Porphyridium Cruentum unicellular algae), a well‐known hydrating, moisturising and a skin barrier repairing agent.

**Objectives:**

To assess the safety, usability and efficacy of SNG100 cream in patients aged ≥6 years with moderate AD.

**Methods:**

In this proof of concept phase I, double‐blind, randomised trial, participants received one of three treatments for 14 days: SNG100 twice daily (BID), hydrocortisone 1% BID or mometasone furoate once daily (QD). The primary endpoint was the safety and tolerability of SNG100 cream compared to hydrocortisone 1% and mometasone furoate. The secondary endpoint was the subject's usability of SNG100. Exploratory efficacy endpoints included percent change from baseline in SCOring AD (SCORAD), Eczema Area and Severity Index, Patient‐Oriented Eczema Measure, Dermatology Life Quality Index, pruritus Numerical Rating Score (NRS), peak pruritus‐NRS and Investigator's Global Assessment. Subjects were also followed up without any treatment for additional 14 days.

**Results:**

Overall, 66 participants were screened, and 60 patients were randomised. SNG100 demonstrated a high safety profile, similar to marketed products hydrocortisone 1% and mometasone furoate 0.1%, with no unanticipated drug safety related events. SNG100 and mometasone furoate 0.1% cream achieved almost similar and statistically significant greater percentage reductions from baseline in SCORAD as compared to hydrocortisone 1% cream. SNG100 demonstrated significant improvement in NRS as compared to hydrocortisone 1% cream. Remarkably, SNG100 led to a lasting effect with only 29.4% of subjects returning to IGA3 during the follow‐up period compared to 50% and 38.9% in the hydrocortisone 1% and in mometasone furoate treatment arms, respectively.

**Conclusions:**

Topical SNG100 is an effective, safe, and well‐tolerated innovative treatment for moderate AD. Trial registration number: NCT04615962 (Topical Cream SNG100 for Treatment in Moderate AD Subjects).

## Graphical abstract

Safe and effective topical therapies to improve disease control and long‐term outcomes in atopic dermatitis (AD) are needed. This clinical trial demonstrates the safety, usability and efficacy of topical SNG100, a novel formulation of hydrocortisone 1% with sulphated polysaccharide (extraction of Porphyridium Cruentum from algae), for moderate AD patients aged 6 years of age or older, as compared to low and mid‐potency corticosteroids, hydrocortisone 1% and mometasone furoate, respectively.

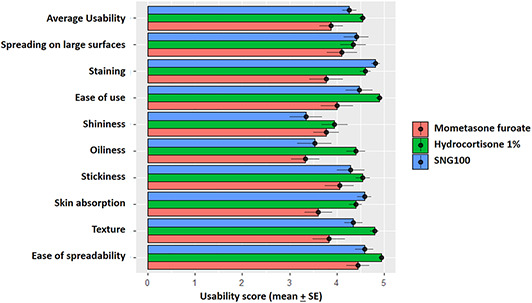

**What is already known about this topic?**

- Safe and effective topical therapies to improve disease control and long‐term outcomes in atopic dermatitis (AD) are needed.
- Although novel topical therapies for AD are under development, topical corticosteroids still serve as first line agents; however, concerns about potential adverse effects limit the use of potent corticosteroids.
- SNG100 is a novel composition of hydrocortisone 1% with sulphated polysaccharide (extraction of Porphyridium Cruentum from algae), a well‐known hydrating, moisturising and a skin barrier repair agent.

**What does this study add?**

- This trial demonstrates the safety, usability and efficacy of topical SNG100 for moderate AD patients aged 6 years of age or older.
- SNG100 offers a novel alternative to current topical treatments for AD.

## 1 INTRODUCTION

Atopic dermatitis (AD) is a common pruritic inflammatory skin disease affecting up to 10% of adults and 20% of children with considerable impact on patients and caregivers quality of life.^1^, ^2^, ^3^, ^4^, ^5^, ^6^ Although considered an immune‐driven disease, AD has a complex pathophysiology, which also involves epidermal defects and impaired bacterial colonisation and diversity.^7^, ^8^ Accordingly, treatment regimens include skin moisturisers, topical immunomodulators (corticosteroids, calcineurin inhibitors, phosphodiesterase 4 inhibitors and JAK inhibitors), phototherapy, systemic immunosuppressive treatments and biologics for moderate‐to‐severe cases.^9^, ^10^ However, there is still an unmet need for safe and effective therapies to improve disease control and long‐term outcomes.^11^ In addition, there is a need for more effective but safer treatments for treating delicate and sensitive skin surfaces (e.g. face, intertriginous areas) with no risk of atrophy following long‐term use.

Corticosteroids are available for treatment of AD by various routes of administration, including topical, oral, and intramuscular. Although systemic corticosteroids result in rapid improvement, their discontinuation results in recurrence of disease of greater severity, restricting their use. Topical corticosteroids (TCS) still represent the mainstay of treatment in mild‐to‐moderate AD but their long‐term use may result in a wide array of complications, especially in children.^12^, ^13^, ^14^

SNG100 is a novel formulation comprising the low‐potency corticosteroid hydrocortisone 1% in combination with sulphated polysaccharides (SPS) extracted from Porphyridium cruentum microalgae, an excellent gelling agent and stabiliser, with well‐known hydrating, moisturising and skin barrier repair capabilities.^15^, ^16^, ^17^ Moreover, SNG100 quickly absorbs and provides a lubricating quality contributing to patient compliance, tolerance, and adherence.

In the current randomised, double‐blinded study, we aimed to assess the safety, tolerability and efficacy of SNG100 in moderate AD patients as compared to marketed products hydrocortisone 1% and mometasone furoate 0.1%.

## 2 METHODS

### 2.1 Study design

This was a proof of concept phase I, multicenter, randomised, double‐blind, parallel study to evaluate the safety and tolerability of topical SNG100 cream in participants with moderate AD (clinicaltrials.gov: NCT04615962). Participants were randomised at 5 sites in Israel between March 2020 and April 2022. The study included a screening visit, a 2‐week treatment period and a 2‐week follow‐up period (see Figure S1 for study design). This study was conducted in compliance with the principles of the Declaration of Helsinki and the study protocol was reviewed and approved by Institutional Review boards at each participating centre. All participants or their legal guardians provided written informed consent.

### 2.2 Participants

Male and female patients, 6 years or older, with a diagnosis of moderate AD, were enroled. At screening and baseline, participants were required to have an Investigator's Global Assessment (IGA) score of 3, Eczema Area and Severity Index (EASI) total score of 7.1–21 and scoring AD (SCORAD) of 26–50. Exclusion criteria included participants with AD restricted to face and scalp, unstable disease requiring consistent treatment with high potency TCS, active secondary infection, uncontrolled diabetes mellitus or autoimmune disease, pregnancy or breastfeeding. In addition, patients were required to discontinue topical medications, systemic treatments/phototherapy and biologics, 2 weeks, 4 weeks and 6 months prior to enrolment, respectively.

### 2.3 Interventions

Participants were randomised to three treatment arms: SNG100 twice daily (BID) (containing 1.0% (w/w) of hydrocortisone in SNG cream vehicle comprising Porphyridium cruentum exopolysaccharides), hydrocortisone 1% cream BID (Hydrocutan crème™, Dermapharm, GmbH) or mometasone furoate 0.1% (ECRUAL Fettcreme™, Organon, France) once daily (QD), for 14 days (Figure S1). The amount of applied topical medication was determined based on patient's age and involved body surface: 2.5 and 4 fingertip units (FTUs) for entire upper extremity in 6–10 and > 10 years of age, respectively; 4.5 and 8 FTUs for entire lower extremity in 6–10 and > 10 years of age, respectively; 3.5 and 7 FTUs for front chest and abdomen in 6–10 and > 10 years of age, respectively; 5 and 7 FTUs for back and buttocks in 6–10 and > 10 years of age, respectively. The medications (of either treatment arm) were applied to all involved skin areas. No emollients were allowed during the study. Investigators were blinded to both treatment and frequency of dosing while participants were blinded to treatment only. Central block randomisation was issued by an external vendor who provided a randomisation number and a treatment kit number for each participant. Prior to baseline, participants and caregivers were instructed to apply the treatment on affected areas excluding the face and the scalp.

### 2.4 Endpoints

The primary endpoints were safety and tolerability, assessed by self‐reporting experience, measuring the incidence of treatment‐emergent adverse events (TEAEs) including serious AEs (defined as medical events which led to death, were life threatening, required hospitalisation or resulted in disability), assessing significant changes (based on physician assessment) in vital signs (blood pressure, heart rate and temperature) and laboratory assessment (urinalysis). Adverse events were reported from screening until the end of the follow up period. The secondary endpoints were usability factors of SNG100 cream versus hydrocortisone 1% and mometasone furoate cream. Usability was evaluated by self‐completion of a 9 questions usability questionnaire completed at visit 3 (see Appendix 1 for usability questionnaire).

To explore the clinical effect of SNG100 novel cream (Hydrocortisone 1% with SPS) versus commercial reference products (hydrocortisone 1% or mometasone furoate 0.1% cream), the total reduction and percentage change from baseline in EASI, SCORing AD (SCORAD), Investigator's Global Assessment (IGA), Patient‐Oriented Eczema Measure (POEM), Dermatology Life Quality Index (DLQI), Numerical Rating Score (NRS) and peak pruritus‐NRS, were assessed at screening and baseline (visits 1 and 2), days 7 and 14 of treatment (visits 3 and 4) and after 14 days follow up (visit 5) (see Table S1 for study protocol).

### 2.5 Statistical analysis

As this was a phase 1 study, sample size determination was not planned to meet specific statistical significance and power requirements. Analyses were based on existing data without any imputations. The intent to treat set was used for safety while the Per Protocol set (PP) was used for efficacy evaluation. Continuous variables were summarised with mean and 95% confidence interval or median and interquartile range (IQR) and compared between study arms using Kruskal Wallis test followed by Dunn's post hoc test. Categorical variables were summarised using frequency and percentages and compared between study arms using Fisher exact test or Chi‐square test. The Friedman's paired test was used to compare each follow‐up time point to baseline within study arms. A general estimating equations (GEE) model was applied to estimate the EASI scores during repeated visits and to compare between 3 treatment arms, accounting for the within‐subject correlation and adjusting for age and gender. A *p*‐value <0.05 was considered statistically significant. Analyses were carried out using R Core Team (2021).

## 3 RESULTS

### 3.1 Participants

In total, 66 subjects were screened out of whom 60 were enroled and randomised to treatment (Figure S1). The mean age of participants was 41.6, 29.5 and 39.1 years in the mometasone furoate, hydrocortisone 1% and SNG100 groups, respectively. Thirty five randomised participants were males (61.4%) and 12 subjects (21%) were under the age of 18. All randomised patients demonstrated a moderate IGA score of 3 at baseline. The mean EASI and SCORAD scores at baseline were 11.3 and 45.6 for all enroled patients, respectively. No significant differences between study groups in baseline DLQI, POEM, pruritus NRS and PP‐NRS were observed. Baseline demographic and disease characteristics stratified by treatment are shown in Table 1. Overall, 55 participants (96.5%) completed treatment according to study protocol. Two patients from the SNG100 group discontinued treatment prior to visit 5 (one as a result of disease flare and one was lost to follow‐up) and were excluded from the exploratory outcome analysis.

**TABLE 1:** Demographics and baseline disease characteristics.

Baseline demographics and disease severity	Mometasone furoate	Hydrocortisone 1%	SNG100	*p* value
Patient enroled^a^	*n* = 18	*n* = 20	*n* = 17	
Age (mean, SD, range)	42.01, 21.10, 7.09–79.36	30.22, 15.99, 8.26–75.12	42.42, 20.43, 10.41–74.82	NS
Gender (F:M)	9:9	7:13	5:12	NS
IGA (%)	3 (100%)	3 (100%)	3 (100%)	NS
EASI (mean, SD, range)	12.21, 4.21, 7.2–18.9	11.66, 4.22, 7.1–18	10.02, 3.54, 7.2–20.7	NS
SCORAD (mean, SD, range)	44.72, 5.09, 31.95–50	46.45, 4.49, 35.25–49.95	45.74, 3.86, 36–50	NS
DLQI (mean, SD, range)	13, 5.73, 3–26	10.65, 6.49, 1–25	9.24, 5.47, 1–18	NS
POEM (mean, SD, range)	17, 7.04, 6–28	18.8, 5.52, 10–28	15.29, 7.57, 5–28	NS
Pruritus‐NRS (mean, SD, range)	7.44, 1.72, 3–10	7.3, 2.41, 2–10	7.12, 1.87, 4–10	NS
PP‐NRS (mean, SD, range)	7.83, 2.15, 2–10	7.9, 2.07, 4–10	8.18, 2.6, 0–10	NS

^a^ Including only patients who completed the study.

Abbreviations: DLQI, dermatology life quality index; EASI, eczema area and severity index; IGA, investigator's global assessment; NRS, numerical rating score; NS, not significant; POEM, patient‐oriented eczema measure; PP‐NRS, Peak pruritus numeric rating score; SCORAD, scoring atopic dermatitis.

### 3.2 Primary outcome: Safety and tolerability

Overall, 5 participants experienced AEs during the study (Table S2). In the SNG100 group, one TEAE with worsening of AD was observed. Another patient experienced SAE with Crohn's disease exacerbation leading to hospitalisation. COVID‐19 infection was observed in another patient in the SNG100 treatment arm. Two additional unrelated AEs were observed in the mometasone furoate and the hydrocortisone 1% groups (see Table S2). Normal physical examination and vital signs were recorded in all participants throughout the study. No clinically meaningful trends in urinalysis were observed.

### 3.3 Secondary outcome: Usability

**FIGURE 1:**
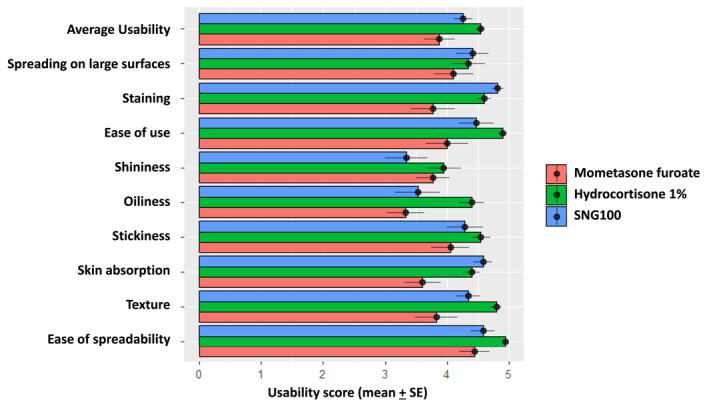
Usability and tolerability. Average usability score and individual usability parameters are presented for SNG100, mometasone furoate and hydrocortisone 1% arms. Each variable was graded from 1 to 5 (higher score = better for use). All scores were averaged (mean + SD) per each treatment arm. See Appendix 1 for usability questionnaire.

Treatment with SNG100 resulted in excellent usability with an average usability score above 4, similar to hydrocortisone 1% (Figure 1 and Table S3). In contrast, mometasone furoate revealed a lower mean usability score (3.88 ± 1.05); however differences were not statistically significant between the 3 treatment arms. Regarding shininess and oiliness, the mean usability scores were below 4 for SNG100; however all participants rated above 4 all other usability parameters (ease of spreading, staining, texture, stickiness, skin absorption) similar to hydrocortisone 1% cream (Figure 1 and Table S3).

### 3.4 Exploratory outcomes

**FIGURE 2:**
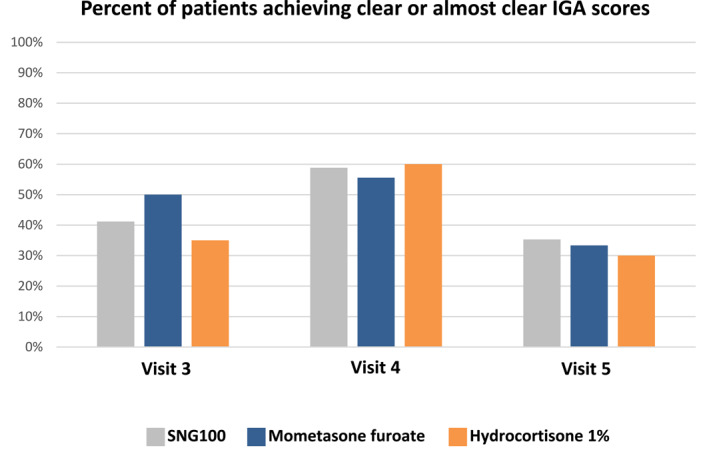
Percent of AD patients with IGA ‘clear’ or ‘almost clear’. The figure represents the percent of subjects that improved as compared to baseline (reached a score of 0 [clear] or 1 [almost clear] at each visit).

Baseline IGA was 3 for all participants (Table 1). On day 7 (visit 3), only 11.8% of patients in the SNG100 arm had IGA 3, while 41% of patients demonstrated IGA scores of 0 (clear) or 1 (almost clear). Additional improvement was seen at day 14 (visit 4) with 5.9% and 58.8% of patients demonstrating IGA of 3 or IGA of 0/1, respectively. Similar results were observed with mometasone furoate (50% and 55.5% achieving IGA 0/1 at visit 3 and 4, respectively) while in the hydrocortisone 1% group, 35% of patients achieved IGA 0/1 at visit 3 while at visit 4, 60% and 20% demonstrated an IGA score of 0/1 and 3, respectively (Table S4 and Figure 2). The results were not statistically significant between the 3 treatment arms. Gradual worsening in IGA was documented in all study arms following treatment discontinuation at visit 5; however, only 29.4% had an IGA score of 3 in the SNG100 arm as compared to 50% and 38.9% in the hydrocortisone 1% and mometasone furoate groups, respectively (Table S4 and Figure S2).

**FIGURE 3:**
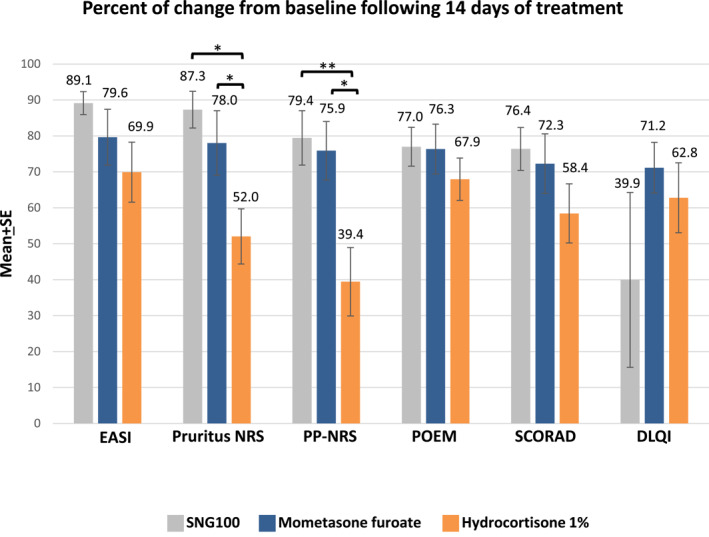
Percentage decrease from baseline in exploratory outcome parameters at day 14 (visit 4). The figure represents the percent change from baseline to day 14 in EASI, pruritus BRS, PP‐NRS, POEM, SCORAD and DLQI. DLQI, dermatology life quality index; EASI, eczema area and severity index; NRS, numerical rating score; POEM, patient‐oriented eczema measure; PP‐NRS, Peak pruritus numeric rating score; SCORAD, scoring atopic dermatitis. *p ≤ 0.05, **p ≤ 0.01.

The mean EASI score at baseline was 12.21, 11.66 and 10 for the mometasone furoate, hydrocortisone 1% cream and SNG100, respectively (Table 1). Across all groups there was a steady and significant trend for improvement in EASI score as compared to baseline, with best results achieved in all study arms at day 14 (visit 4) (Figure 3). In addition, a steady improvement in percentage of change from baseline in EASI score was observed, with 69.9%, 79.64% and 89.13% improvement from baseline in the hydrocortisone 1%, mometasone furoate and SNG100 groups, respectively, at visit 4. No statistically significant differences between groups were noted (Table S4). Based on the GEE model, the SNG100 group demonstrated significantly lower EASI score values as compared to mometasone furoate (−1.76 *p* = 0.040) and hydrocortisone 1% cream (−1.77, *p* = 0.042) (Table S5). No significant differences were noted between mometasone furoate and hydrocortisone 1% cream. Of note, although a slight increase in EASI score was observed in all 3 groups at visit 5, results were still lower than baseline, with lowest values observed in the SNG100 group as compared to the 2 other treatment arms (Table S4).

Similar to EASI score, a statistically significant reduction in SCORAD score was observed in all treatment groups (best results achieved at visit 4) with no significant difference in SCORAD change observed between groups (Table S4 and Figure 3). A steady improvement in percentage of change from baseline in SCORAD score was noted, with 58.43%, 72.29% and 76.38% mean improvement from baseline observed in the hydrocortisone 1%, mometasone furoate and SNG100 groups, respectively, at visit 4 (Figure 3). Although not statistically significant, approximately 60% of patients in the mometasone furoate and SNG100 groups achieved a SCORAD change ≥75% (SCORAD75), as compared to only 40% in the hydrocortisone 1% arm at week 4 (Table S6). Although an increase in SCORAD score was noted at follow‐up (visit 5), the results were still lower than baseline, with lowest values observed in the SNG100 group similar to what had been observed with the EASI score (Table S4).

In all arms, a significant reduction in DLQI scores was noted with best results achieved at visit 4 (Table S4). At visit 5, the lowest DLQI score was achieved in the SNG100 arm (4.12), followed by mometasone furoate and hydrocortisone 1% cream (5.44 and 7.63, respectively). At visit 4, a median change in DLQI ranged from 71.1% in the hydrocortisone 1% arm to 77.8% in the SNG100 arm. A significant improvement in DLQI score was noted between visits 3 and 4 in the SNG100 arm (53.3%–77.8%, *p* < 0.01). There was no significant difference in percent of change from baseline of DLQI between groups at all visits (Figure 3).

In all arms, a significant reduction in POEM score was noted, with best results achieved at visit 4 (Table S4). No significant difference in total or percentage of change from baseline in POEM score was noted between the 3 groups (Figure 3). At visit 5, best results were achieved with SNG100 (53.86% change in mean POEM score), followed by mometasone furoate and hydrocortisone 1% cream (48.57% and 33.3%, respectively). At visit 4, median change in POEM ranged from 74.3% in hydrocortisone 1% arm, 83.33% in the SNG100 arm and 86.6% in the mometasone furoate arm.

A significant reduction in pruritus NRS and PP‐NRS scores was noted in all arms, with best results achieved at visit 4. For pruritus NRS, SNG100 and mometasone furoate arms had significantly lower values as compared to hydrocortisone 1% (*p* = 0.018 and *p* = 0.020, respectively, Dunn's Post hoc test). Similarly, also for PP‐NRS, SNG100 and mometasone furoate arms had significantly lower values as compared to hydrocortisone 1% (*p* = 0.007 and *p* = 0.013, respectively, Dunn's Post hoc test). At visit 4, a significant change in mean pruritus NRS and PP‐NRS was noted (Figure 3) and ranged from 52% to 39.43% in the hydrocortisone 1% arm to 87.3% and 79.44% in the SNG100 arm (*p* < 0.01), respectively.

## 4 DISCUSSION

This double‐blind, randomized study supports the safety and efficacy of the topical SNG100 formulation for moderate AD. SNG100, which is a novel formulation comprising hydrocortisone 1% and SPS, showed advantage in efficacy over hydrocortisone 1% cream and comparable non‐inferior efficacy to the mid‐potency TCS, mometasone furoate 0.1% cream.

The current therapeutic options for AD range from topical treatments for mild‐to‐moderate disease, to phototherapy, systemic and biologic agents for moderate‐to‐severe cases.^2^, ^11^, ^18^, ^19^ Topical treatments include TCS, calcineurin, phosphodiesterase 4 and Janus kinase inhibitors, and additional topical non‐steroidal formulations are currently under development.^20^, ^21^, ^22^ TCS, given their significant anti‐inflammatory effects, still serve as first line topical treatment in most cases of AD; however, although effective, these medications can lead to adverse effects following prolonged and inadequate use, including skin atrophy, telangiectasias and striae.^23^, ^24^ In addition, given the chronic nature of AD, many patients require prolonged and repetitive use of TCS, which further expose them to potential side effects due to systemic absorption, especially with the use of mid‐to‐high potency formulations. TCS treatment is further complicated with ‘steroid phobia’, prevailing adequate treatment with TCS in many AD cases.^25^, ^26^, ^27^ Accordingly, appropriate choice of TCS potency/vehicle and detailed instructions of use including restricted application are required to optimise patient's compliance and to minimise AEs. Taken together, there is a critical need to identify TCS formulations harbouring high efficacy but with lower risk for side effects, allowing prolonged and safe use and that can be safely applied over gentle skin surfaces.

The results of the current study show that SNG100 demonstrates similar efficacy to mometasone furoate 0.1%, albeit the fact that the steroid component in SNG100, hydrocortisone 1%, is a low‐potency steroid. All exploratory outcome measures including physician‐reported clinical signs of AD and the patient‐reported symptoms outcomes, demonstrated improvement following 7 days of treatment with further benefit observed at day 14 of treatment. The average improvement in the SNG100 group as compared to baseline at day 14 was 89%, 87%, 79%, 77%, 76% and 39% in EASI, pruritus‐NRS, PP‐NRS, POEM, SCORAD and DLQI, respectively. Moreover, for most efficacy outcomes, SNG100 led to best improvement, followed by mometasone furoate, with hydrocortisone 1% cream being the least effective. In addition, SNG100 exerted a relative persistent effect on EASI score at visit 5 (14 days following treatment discontinuation) with 69% of patients demonstrating improvement of AD severity as compared to baseline. This is in contrast to only 54% and 42% of patients in the mometasone furoate and hydrocortisone 1% cream groups, respectively, demonstrating improvement in EASI score at visit 5. Similarly, clinically relevant reduction in average pruritus was observed as early as day 7 in the SNG100 group with further improvement at day 14 (69% and 87%, respectively) and persistent effect at 14 days follow‐up observed in 40% of patients (as compared to 29% and 25% in the mometasone furoate and hydrocortisone 1% groups, respectively).

Although preliminary, these observations point to a possible long‐term immunomodulatory and anti‐pruritic effect of SNG100. Accordingly, SNG100 containing the low potency TCS hydrocortisone 1% may serve as an effective topical treatment for AD patients with very low risk for TCS‐associated cutaneous side effects and possible superior proactive and maintenance activity as compared to other currently available topical treatments for AD given its potential long‐term effect on the immune system. In addition, given the comparable non‐inferior efficacy of SNG100 to the mid‐potency corticosteroid mometasone furoate 0.1%, SNG100 can be safely applied on gentle skin surfaces with no risk of long term side effects given its low potency steroid content.

SNG100 formulation was well‐tolerated with usability score similar to hydrocortisone 1% and superior to mometasone furoate 0.1% marketed products. In addition, only one reported case of AD worsening was observed with SNG100.

A limitation of this study is its relatively small sample size. Given the known beneficial effect of TCS in AD, to detect significant differences between treatment arms, larger group sizes are needed. In addition, the treatment period spanned only 2 weeks. Formal efficacy assessment over a longer treatment and follow‐up periods would be required to confirm persistent efficacy and long‐term safety profile. This study's strengths included monotherapy without any rescue therapy providing added robustness to the results. In addition, the patient‐reported outcomes strongly supported the clinical improvement shown by objective measures with SNG100 treatment.

In conclusion, the results of this study showed that SNG100 formulation is effective and well tolerated in moderate AD. Further studies are required to evaluate SNG100 safety and efficacy in a larger sample size and for a longer treatment period.

## CONFLICT OF INTEREST STATEMENT

L.S. has served as principal Investigator and/or sub‐Investigator for Eli Lilly, Abbvie, Pfizer, and has participated in advisory boards of Abbvie, Pfizer and Sanofi; S.G. has served as principal Investigator and/or sub‐Investigator for Eli Lilly, Abbvie, Pfizer and Amgene and also received honoraria and consultancy fees from Eli Lilly, Abbvie and Pfizer, Dexcel, Pierre Fabre and Padagis; R.D‐G. serves as consultant for Pfizer, Janssen, Sanofi, AbbVie, Novartis, La Roche‐Posay, Dexcel, Eli Lilly, Mii Labs and Padagis and served as principal Investigator and/or sub‐Investigator for Abbvie, Sanofi, Mii Labs and Regeneron; Y.R. serves as the CMO of Mii Labs, which develops SNG100, and has also received honoraria and consultancy fees from Eli Lilly, Abbvie, Novartis, Janssen, Neopharm, Giuliani S.p.A, Dexcel Pharma, Sanofi, Taro, Monasterium laboratories and Pfizer; E.S. serves as consultant for Pierre Fabre, Kamari, Biomx, Bayer, Amryt, Medison Pharma, Sol‐Gel, Krystal, BiondVax, Galmed, LaserTeam; I.Z., D.F. and O.Y.Z. work at Synergy dermatology Ltd.

## AUTHOR CONTRIBUTIONS

**Liat Samuelov**: Investigation (lead); methodology (equal); writing – original draft (lead). **Avner Shemer**: Investigation (equal); writing – review & editing (supporting). **Shoshana Greenberger**: Investigation (equal); writing – review & editing (supporting). **Inbal Ziv**: Conceptualization (equal); data curation (equal); formal analysis (equal); methodology (equal); resources (equal); validation (equal); writing – review & editing (supporting). **Doron Friedman**: Conceptualization (lead); data curation (lead); formal analysis (lead); funding acquisition (lead); investigation (equal); methodology (lead); resources (lead); validation (lead); writing – review & editing (equal). **Oron Yacoby‐Zeevi**: Conceptualization (equal); data curation (equal); formal analysis (equal); methodology (equal); resources (equal); validation (equal); writing – review & editing (supporting). **Roni Dodiuk‐Gad**: Investigation (equal); writing – review & editing (supporting). **Yuval Ramot**: Investigation (equal); methodology (equal); writing – review & editing (equal). **Sari Murad**: Investigation (equal); writing – review & editing (supporting). **Eli Sprecher**: Investigation (lead); methodology (lead); writing – original draft (lead).

## ETHICS STATEMENT

This study was conducted in compliance with the principles of the Declaration of Helsinki and the study protocol was reviewed and approved by Institutional Review boards at each participating centre.

## Supporting information

Supporting Information S1

Supporting Information S2

## Data Availability

The data that support the findings of this study are available from the corresponding author upon reasonable request.
