# Supplementary material for: SNG100, a novel topical treatment for moderate atopic dermatitis, in patients aged 6 years or older: A randomised, double‐blind, active‐controlled trial
Source: Skin Health Dis. 2023 Oct 14;3(6):e293. doi: 10.1002/ski2.293 (PMC10690700; doi:10.1002/ski2.293)
Supplement: Supplementary file 1 — Supporting Information S1 [file SKI2-3-e293-s002.docx]

**SNG100, a novel topical treatment for moderate atopic dermatitis, in patients aged 6 years or older: a randomized, double-blind, active-controlled trial**

Liat Samuelov^1,2^, Avner Shemer^2,3^, Shoshana Greenberger^2,4^, Inbal Ziv^5^, Doron Friedman^5^, Oron Yacoby-Zeevi^5^, Roni Dodiuk-Gad^6,7,8^, Yuval Ramot^9,10^, Sari Murad^9,11^ and Eli Sprecher^1,2^

^1^Division of Dermatology, Tel Aviv Sourasky Medical Center, Tel Aviv, Israel; ^2^Faculty of Medicine, Tel Aviv University, Tel Aviv, Israel; ^3^Department of Dermatology, Sheba Medical Center, Tel-Hashomer, Tel Aviv University, Ramat-Gan, Israel; ^4^Department of Dermatology, Pediatric Dermatology Unit, Sheba Medical Center, Ramat Gan, Israel; ^5^Seanergy dermatology, Rehovot, Israel; ^6^Dermatology and Venereology Department, Emek Medical Center, Afula, Israel; ^7^Ruth and Bruce Rappaport Faculty of Medicine, Technion Institute of Technology, Haifa, Israel; ^8^Division of Dermatology, Department of Medicine, University of Toronto, Toronto, Ontario, Canada; ^9^Department of Dermatology, Hadassah-Hebrew University Medical Center, Jerusalem, Israel; ^10^Faculty of Medicine, Hebrew University of Jerusalem, Jerusalem, Israel; ^11^Dermatology Unit, Kaplan Medical Center, Rehovot, Israel

**Supplementary figures**

*Supplementary Figure 1: Study design, flow chart*


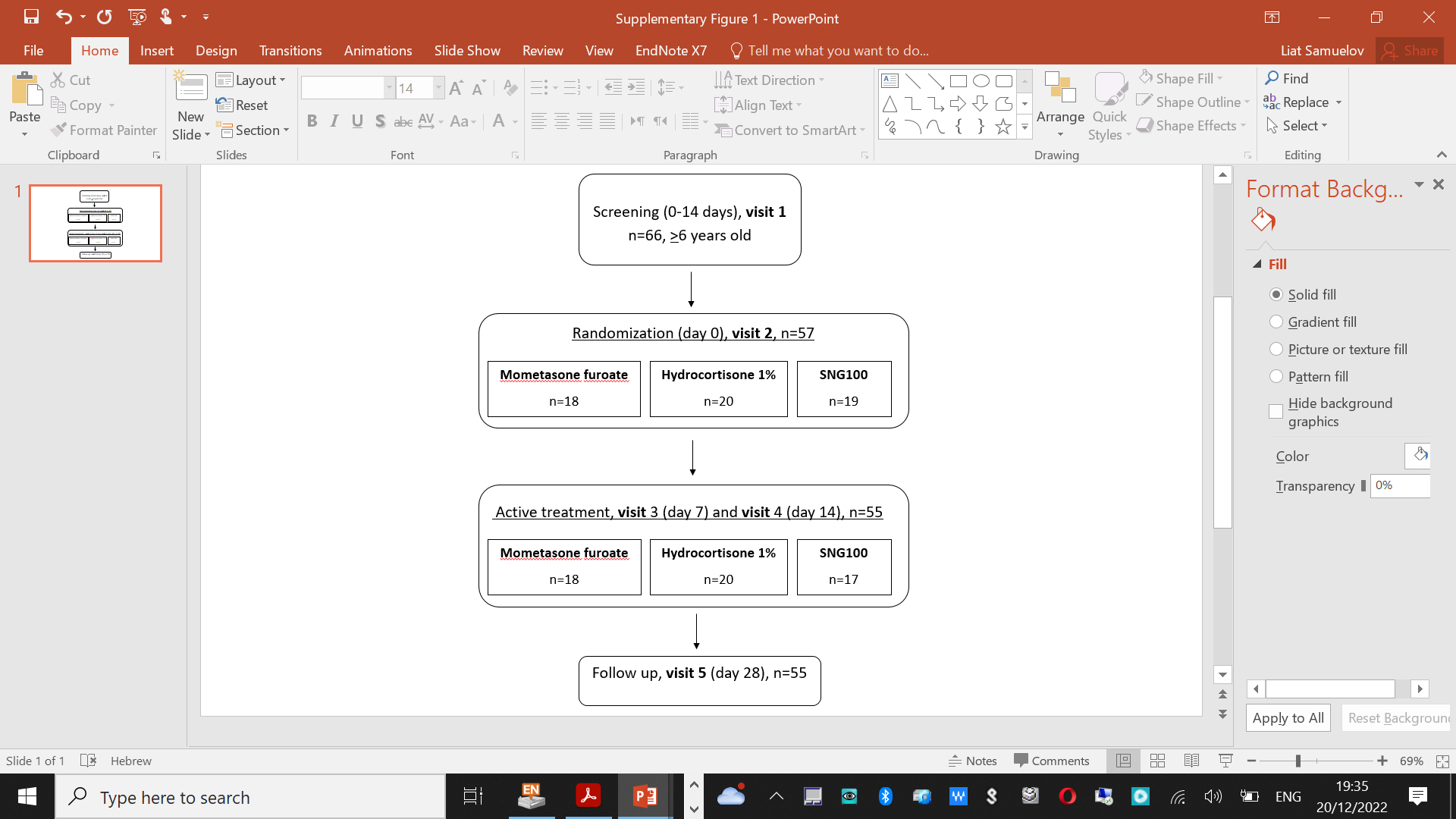


Randomization (day 0), **visit 2**, n=60

n=19

n=21

n=20

*Supplementary Figure 2: Clinical improvement with SNG100 treatment*

*
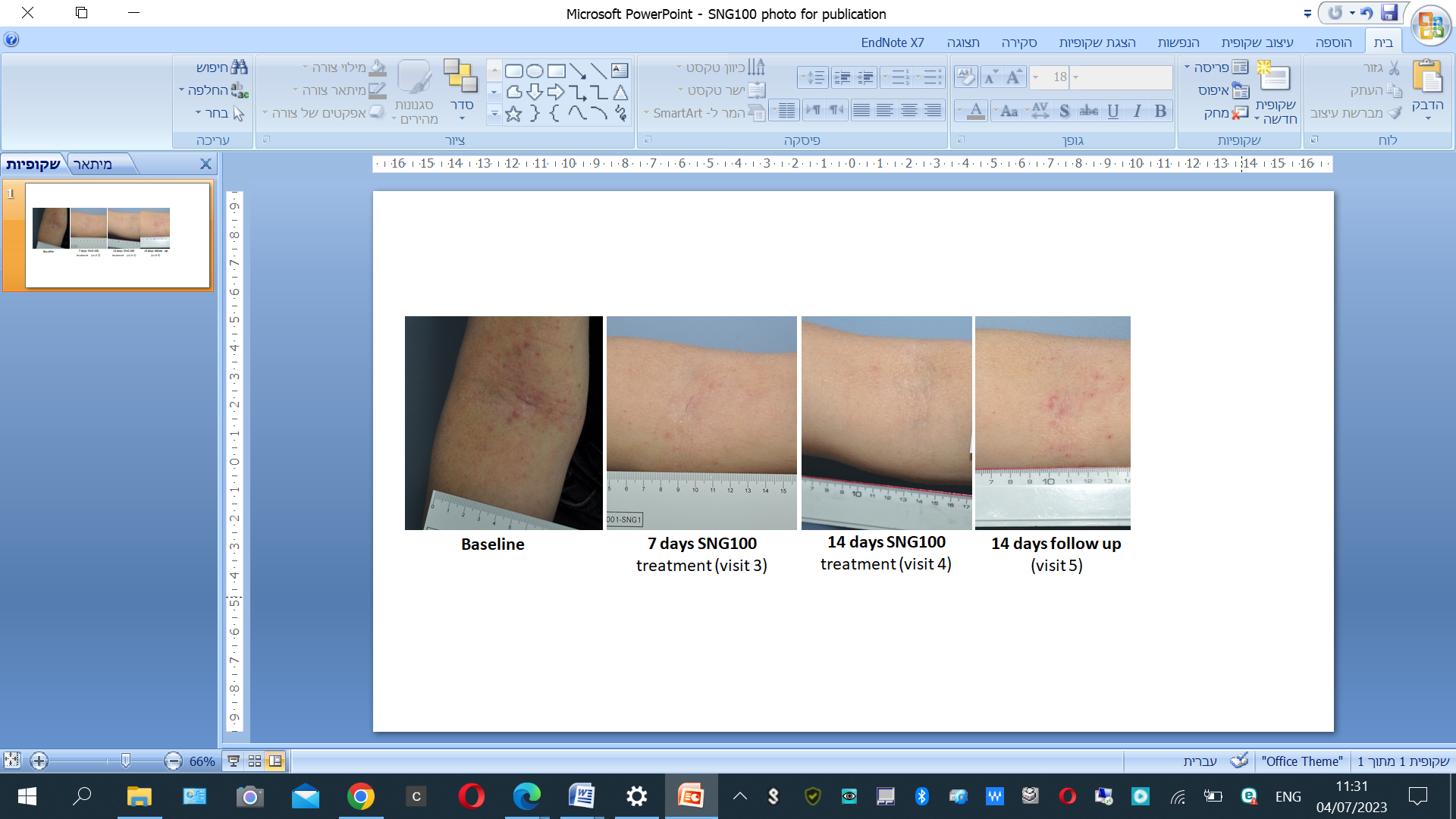
*

Adult patient with atopic dermatitis randomized to the SNG100 treatment arm, demonstrating Investigator's Global Assessment (IGA) score of 3 at baseline with significant improvement in disease severity following 7 and 14 days of treatment. Slight worsening is observed 14 days following treatment discontinuation.

**Supplementary tables**

**Supplementary table 1: study protocol**

| **Visit 1**  **Screening (up to 14 days)** | **Visit 2**  **Day 0 (baseline)*** | **Visit 3**  **Day 7 (treatment)** | **Visit 4**  **Day 14 (treatment)** | **Visit 5**  **Day 28 (follow up)**** |
| --- | --- | --- | --- | --- |
| -Informed consent  -Inclusion and exclusion criteria  -Review of demographics, medical and medication history  -Baseline signs and symptoms  -Physical examination including weight, height, vital signs  -AD clinical assessment:   - IGA - EASI - SCORAD - DLQI - POEM - Pruritus-NRS - PP-NRS   -Urinalysis  -Pregnancy test  -Photographs | -Inclusion and exclusion criteria  -Concomitant medications  -Signs and symptoms and adverse events  -Vital signs  -AD clinical assessment:   - IGA - EASI - SCORAD - DLQI - POEM - Pruritus-NRS - PP-NRS   -Treatment randomization  - First administration of study cream on site  -Providing additional cream tubes and usage instructions  -Pregnancy test  -Photographs | - AEs / SAEs review  -Concomitant medications  -Vital signs  -AD clinical assessment:   - IGA - EASI - SCORAD - DLQI - POEM - Pruritus-NRS - PP-NRS   - Treatment dispensing and accountability  - Usability questionnaire  -Photographs | - AEs / SAEs review  -Concomitant medications  - Physical examination including weight, height, vital signs  -AD clinical assessment:   - IGA - EASI - SCORAD - DLQI - POEM - Pruritus-NRS - PP-NRS   - Treatment return and accountability  -Urinalysis  -Pregnancy test  -Photographs | - AEs / SAEs review***  -Concomitant medications  - Physical examination including weight, height, vital signs  -AD clinical assessment:   - IGA - EASI - SCORAD - DLQI - POEM - Pruritus-NRS - PP-NRS   - Treatment return and accountability (if not returned at visit 4)  -Urinalysis  -Photographs |

*In case the randomization (visit 2) was conducted within 3 days of screening (visit 1) there was no requirement to repeat atopic dermatitis assessment questionnaires and to perform pregnancy test and lesion digital photography.

** During the follow-up period the subjects were instructed to use moisturizing cream advised by the investigator.

*** New SAEs were recorded only during the follow-up period. New adverse events were recorded up to 1-week post visit 4. Follow up of on-going adverse events continued until resolution or until no longer medically indicated per principal investigator.

AD - atopic dermatitis, IGA - investigator's global assessment, EASI - eczema area and severity index, SCORAD - scoring atopic dermatitis, DLQI - dermatology life quality index, POEM - patient-oriented eczema measure, NRS - numerical rating score, PP-NRS - Peak pruritus numeric rating score, AE - adverse event, SAE - serious adverse event

**Supplementary table 2: adverse events summary**

|  | Mometasone furoate  (N=19) | Hydrocortisone 1% (N=21) | SNG100  (N=20) | p value |
| --- | --- | --- | --- | --- |
| SAE | 0 (0%) | 0 (0%) | 1 (5.0%)  Crohn's disease exacerbation | p>0.05 |
| Any AE | 1 (5.2%)  *Menstrual pain* | 1 (4.7%)  *Eye infection* | 1 (5.0%)  *COVID-19 infection* | p>0.05 |
| Related AE | 0 (0%) | 0 (0%) | 1 (5.0%)  *Worsening of atopic dermatitis* | p>0.05 |

**Supplementary table 3: usability and tolerability outcome**

| Usability and tolerability parameters | Mometasone furoate | Hydrocortisone 1% | SNG100 | p value |
| --- | --- | --- | --- | --- |
|  | **n=18** | **n=20** | **n=17** |  |
| Easy to spread (mean, median, range) | 4.444, 5, 2-5 | 4.95, 5, 4-5 | 4.588, 5, 3-5 | p>0.05 |
| Texture (mean, median, range) | 3.833, 4.5, 1-5 | 4.8, 5, 4-5 | 4.353, 5, 3-5 | p>0.05 |
| Skin absorption (mean, median, range) | 3.611, 4, 1-5 | 4.4, 4, 3-5 | 4.588, 5, 3-5 | p>0.05 |
| Stickiness (mean, median, range) | 4.056, 5, 1-5 | 4.550, 5, 3-5 | 4.294, 5, 1-5 | p>0.05 |
| Oiliness (mean, median, range) | 3.333, 3, 1-5 | 4.4, 5, 2-5 | 3.529, 4, 1-5 | p>0.05 |
| Shininess (mean, median, range) | 3.778, 4, 1-5 | 3.950, 4, 1-5 | 3.353, 3, 1-5 | p>0.05 |
| Ease of use (mean, median, range) | 4, 5, 1-5 | 4.9, 5, 4-5 | 4.471, 5, 1-5 | p>0.05 |
| Staining (mean, median, range) | 3.778, 4, 1-5 | 4.6, 5, 4-5 | 4.824, 5, 4-5 | p>0.05 |
| Spreading on large surface (mean, median, range) | 4.11, 5, 1-5 | 4.350, 5, 1-5 | 4.412, 5, 1-5 | p>0.05 |
| Average usability (mean, median, range) | 3.88, 4.22, 1.44-5 | 4.54, 4.56, 3.89-5 | 4.27, 4.56, 2.89-5 | p>0.05 |

**Supplementary table 4: efficacy assessment using exploratory outcome measures**

| Visit | Mometasone furoate | Hydrocortisone 1% | SNG100 | p value |
| --- | --- | --- | --- | --- |
| IGA | | | | |
| Visit 3 n=18 n=20 n=17 | | | | |
| 0  1  2  3 | 3 (16.7%)  6 (33.3%)  7 (38.9%)  2 (11.1%) | 1 (5.0%)  6 (30.0%)  11 (55.0%)  2 (10.0%) | 5 (29.4%)  2 (11.8%)  8 (47.1%)  2 (11.8%) | NS |
| Visit 4 n=18 n=20 n=17 | | | | |
| 0  1  2  3 | 8 (44.4%)  2 (11.1%)  7 (38.9%)  1 (5.6%) | 7 (35.0%)  5 (25.0%)  4 (20.0%)  4 (20.0%) | 7 (41.2%)  3 (17.6%)  6 (35.3%)  1 (5.9%) | NS |
| Visit 5 n=18 n=20 n=17 | | | | |
| 0  1  2  3  4 | 4 (22.2%)  2 (11.1%)  4 (22.2%)  7 (38.9%)  1 (5.6%) | 2 (10.0%)  4 (20.0%)  4 (20.0%)  10 (50.0%)  0 (0.0%) | 4 (23.5%)  2 (11.8%)  6 (35.3%)  5 (29.4%)  0 (0.0%) | NS |
| EASI | | | | |
| Visit 3 n=18 n=20 n=17 | | | | |
| *EASI values*  Mean (SD), median (Q1, Q3)  *EASI % change from baseline*  Mean (SD), median (Q1, Q3) | 3.37 (3.31), 2.1 (0.52, 6.15)^*^  71.6 (27.85), 80.11 (55.11, 95.51) | 3.66 (3.4), 3.3 (0.75, 5.25)^*^  66.17 (31.41), 71.54 (54.71-94.66) | 3.22 (3), 3.2 (0.4, 4.4)^*^  67.61 (29.79), 69.62 (54.55, 94.44) | NS  NS |
| Visit 4 n=18 n=20 n=17 | | | | |
| *EASI values*  Mean (SD), median (Q1, Q3)  *EASI % change from baseline*  Mean (SD), median (Q1, Q3) | 2.42 (4.04), 0.35 (0, 3.07)^*^  79.64 (32.87), 95.14 (69.35, 100) | 3.11 (3.57), 1.8 (0, 5.65)^*^  69.9 (37.41), 84.2 (55.71, 100) | 1.21 (1.78), 0.8 (0-2)^*^  89.13 (13.13), 92.45 (83.33, 100) | NS  NS |
| Visit 5 n=18 n=20 n=17 | | | | |
| *EASI values*  Mean (SD), median (Q1, Q3)  *EASI % change from baseline*  Mean (SD), median (Q1, Q3) | 5.39 (5.94), 3.45 (1.15, 8.1)  54.12 (46.38), 71.39 (18.12, 90.13) | 6.60 (5.99), 5.95 (1.73, 10.8)  41.98 (49.3), 53.12 (-1.41, 81.99) | 2.94 (2.79), 2.6 (0.4, 4.7)  68.96 (31.52), 76.14 (58.62, 94.94) | NS  NS |
| SCORAD | | | | |
| Visit 3 n=18 n=20 n=17 | | | | |
| *SCORAD values*  Mean (SD), median (Q1, Q3)  *SCORAD % change from baseline*  Mean (SD), median (Q1, Q3) | 20.91 (14.38), 20.93 (10.21, 26.22)^*^  53.8 (30.3), 54.9 (36.72, 78.74) | 23.79 (13.17), 25.9 (14.97, 34.25)^*^  48.65 (28.13), 43.94 (24.87-69.66) | 18.26 (14.82), 19 (6.85, 27.5)^*^  60.32 (31.91), 58.76 (44.33, 86.08) | NS  NS |
| Visit 4 n=18 n=20 n=17 | | | | |
| *SCORAD values*  Mean (SD), median (Q1, Q3)  *SCORAD % change from baseline*  Mean (SD), median (Q1, Q3) | 12.74 (16.59), 6.25 (0.25, 17.35)  72.29 (35.13), 85.82 (63.71, 99.44)^@^ | 18.91 (16.4), 16.73 (2.88, 33.62)^*^  58.43 (36.74), 63.68 (31.53, 92.99) | 10.74 (11.2), 5.75 (0-21.5)^*^  76.38 (24.65), 87.06 (55.53, 100)^#^ | NS  NS |
| Visit 5 n=18 n=19 n=17 | | | | |
| *SCORAD values*  Mean (SD), median (Q1, Q3)  *SCORAD % change from baseline*  Mean (SD), median (Q1, Q3) | 6.32 (21.03), 24 (8.54, 43.71)  40.13 (48.34), 47.67 (2.28, 79.57) | 32.92 (19.06), 39 (19.35, 48)  29.57 (40.41), 16.81 (2.54, 60.7) | 25.41 (19.16), 27.75 (4.4, 44.5)  44.48 (41.24), 37.57 (7.29, 90.6) | NS  NS |
| DLQI | | | | |
| Visit 3 n=18 n=20 n=17 | | | | |
| *DLQI values*  Mean (SD), median (Q1, Q3)  *DLQI % change from baseline*  Mean (SD), median (Q1, Q3) | 5.28 (4.01), 5.00 (2.25, 7.75)^*^  55.39 (30.56), 59.09 (33.33, 79.74) | 4.90 (4.55), 3.00 (2.00, 7.25)^*^  51.43 (27.97), 55.00 (35.33, 67.50) | 5.24 (2.99), 5.00 (3.00, 7.00)^^^  24.05 (69.22), 53.33 (5.00, 61.81)^$^ | NS  NS |
| Visit 4 n=18 n=20 n=17 | | | | |
| *DLQI values*  Mean (SD), median (Q1, Q3)  *DLQI % change from baseline*  Mean (SD), median (Q1, Q3) | 3.17 (3.35), 2.00 (0.25, 5.50)^*^  71.16 (29.88), 77.35 (63.22, 98.61) | 3.20 (2.89), 2.00 (1.00, 5.00)^*^  62.78 (43.46), 71.11 (57.21, 87.85) | 3.06 (3.29), 2.00 (1.00, 4.00)^*^  39.06 (96.70), 77.78 (32.69, 91.67) | NS  NS |
| Visit 5 n=18 n=19 n=16 | | | | |
| *DLQI values*  Mean (SD), median (Q1, Q3)  *DLQI % change from baseline*  Mean (SD), median (Q1, Q3) | 5.44 (5.90), 3.00 (1.00, 9.50)^*^  49.46 (55.59), 73.08 (10.00, 92.92) | 7.63 (5.33), 6.00 (3.50, 11.00)^^^  13.66 (67.86), 33.33 (-12.50, 61.33)^$^ | 4.12 (3.77), 2.50 (1.75, 6.25)^*^  34.62 (81.36), 67.50 (11.54, 84.62) | NS  NS |
| POEM | | | | |
| Visit 3 n=18 n=20 n=17 | | | | |
| *POEM values*  Mean (SD), median (Q1, Q3)  *POEM % change from baseline*  Mean (SD), median (Q1, Q3) | 7.17 (6.76),6.00 (3.00, 9.50)  53.91 (41.89), 66.23 (28.21, 84.60) | 8.05 (6.66), 5.50 (3.00, 11.75)^*^  60.91 (25.21), 70.00 (44.17, 80.31) | 5.88 (5.45), 4.00 (3.00, 8.00)^*^  63.81 (24.67), 64.71 (50.00, 83.33) | NS  NS |
| Visit 4 n=18 n=20 n=16 | | | | |
| *POEM values*  Mean (SD), median (Q1, Q3)  *POEM % change from baseline*  Mean (SD), median (Q1, Q3) | 3.78 (5.47)*, 1.50 (1.00, 3.75)*^*^  *76.33 (29.39), 86.61 (67.31, 94.36)*^&^ | 6.55 (6.23), *4.50 (2.00, 12.25)*^*^  *67.94 (26.43)***,** *74.29 (45.45, 90.00)* | 3.88 (5.28), *2.00 (1.00, 4.00)*^*^  *77.00 (22.29), 83.33 (64.29, 92.84)* | NS  NS |
| Visit 5 n=18 n=20 n=17 | | | | |
| *POEM values*  Mean (SD), median (Q1, Q3)  *POEM % change from baseline*  Mean (SD), median (Q1, Q3) | 7.39 (7.13)*, 5.00 (2.25, 8.75)*^*^  *48.57 (47.24), 62.61 (3.12, 89.29)* | 11.55 (7.08), *12.00 (5.75, 16.00)*^*^  *33.32 (47.87), 42.86 (7.50, 64.91)* | 7.53 (7.73), *5.00 (2.00, 12.00)*^*^  *53.86 (38.01)*, *61.11 (28.57, 84.62)* | NS  NS |
| Pruritus - NRS | | | | |
| Visit 3 n=18 n=20 n=17 | | | | |
| *Pruritus-NRS values*  Mean (SD), median (Q1, Q3)  *Pruritus % change from baseline*  Mean (SD), median (Q1, Q3) | 3.11 (2.81), 3.50 (0.25, 5.00)^*^  58.34 (37.23), 56.35 (28.57, 97.22) | 2.55 (2.26), 2.00 (1.00, 4.25)^*^  62.98 (32.79), 70.00 (39.38, 90.00) | 2.12 (2.26), 2.00 (0.00, 3.00)^*^  68.11 (39.72), 77.78 (60.00, 100.00) | NS  NS |
| Visit 4 n=18 n=20 n=17 | | | | |
| *Pruritus-NRS values*  Mean (SD), median (Q1, Q3)  *Pruritus % change from baseline*  Mean (SD), median (Q1, Q3) | 1.67 (2.77), 0.00 (0.00, 2.00)^*^  78.02 (38.06), 100.00 (73.02, 100.00) | 3.40 (2.58), 3.00 (1.00, 5.25)^*^  52.02 (34.36), 50.00 (28.75, 83.93) | 0.82 (1.24), 0.00 (0.00, 1.00)^*^  87.31 (21.02), 100.00 (80.00, 100.00) | <0.01  <0.01 |
| Visit 5 n=18 n=20 n=17 | | | | |
| *Pruritus-NRS values*  Mean (SD), median (Q1, Q3)  *Pruritus % change from baseline*  Mean (SD), median (Q1, Q3) | 4.33 (4.04), 4.00 (0.00, 8.00)^^^  29.07 (78.51), 45.54 (-13.49, 100.00)^$^ | 5.25 (3.19), 5.00 (2.75, 7.25)^~^  25.13 (43.68), 25.40 (-4.17, 52.50)^&^ | 4.24 (3.60), 4.00 (1.00, 8.00)^^^  40.40 (50.42), 55.56 (0.00, 83.33) ^^^ | NS  NS |
| Peak pruritus – NRS | | | | |
| Visit 3 n=18 n=20 n=17^**^ | | | | |
| *PP-NRS values*  Mean (SD), median (Q1, Q3)  *PP-NRS % change from baseline*  Mean (SD), median (Q1, Q3) | 3.78 (3.56), 4.00 (0.00, 6.00)^*^  54.35 (41.56), 55.00 (16.96, 100.00) | 3.70 (3.06), 3.00 (1.75, 7.00)^*^  50.97 (45.11), 60.00 (23.75, 82.50) | 3.41 (3.69), 2.00 (0.00, 5.00)^*^  60.41 (45.34), 83.75 (35.00, 100.00) | NS  NS |
| Visit 4 n=18 n=20 n=17^**^ | | | | |
| *PP-NRS values*  Mean (SD), median (Q1, Q3)  *PP-NRS % change from baseline*  Mean (SD), median (Q1, Q3) | 2.06 (3.04), 0.50 (0.00, 2.75)^*^  75.89 (34.47), 95.00 (66.32, 100.00) | 4.60 (3.23), 5.00 (2.00, 6.25)^*^  39.43 (42.49), 33.75 (18.75, 73.02) | 1.59 (2.67), 0.00 (0.00, 2.00)^*^  79.44 (31.11), 100.00 (66.25, 100.00) | <0.01  <0.01 |
| Visit 5 n=18 n=20 n=17^**^ | | | | |
| *PP-NRS values*  Mean (SD), median (Q1, Q3)  *PP-NRS % change from baseline*  Mean (SD), median (Q1, Q3) | 4.61 (4.05), 4.00 (0.25, 8.75)^^^  31.64 (63.77), 42.06 (-12.15, 96.88)^$^ | 5.85 (3.27), 6.50 (3.00, 9.00)  21.61 (48.25), 13.39 (0.00, 64.38) | 5.12 (3.53), 5.00 (2.00, 8.00)^^^  40.53 (43.23), 38.75 (0.00, 81.43)^$^ | NS  NS |

^*^Statistically significant results as compared to visit 1, per treatment arm (p<0.001), ^@^ Statistically significant results as compared to visit 1, per treatment arm (p<0.01), ^#^ Statistically significant results as compared to visit 1, per treatment arm (p<0.05), ^$^ Statistically significant results as compared to visit 4, per treatment arm (p<0.01), ^&^ Statistically significant results as compared to visit 3, per treatment arm (p<0.01), ^^^ Statistically significant results as compared to visit 4, per treatment arm (p<0.001), ^~^ Statistically significant results as compared to visit 3, per treatment arm (p<0.001), ^**^ In the SNG100 group *PP-NRS % change from baseline* analysis, only 16 patients were included, IGA - investigator's global assessment, EASI - eczema area and severity index, SCORAD - scoring atopic

dermatitis, DLQI - dermatology life quality index, POEM - patient-oriented eczema measure, NRS - numerical rating score, PP-NRS - Peak pruritus numeric rating score, NS – not significant

**Supplementary Table 5: Eczema area and severity index (EASI) results based on the general estimating equations (GEE) model**

| Arm | Arm | Mean  Difference | P-value | 95% CI | |  |
| --- | --- | --- | --- | --- | --- | --- |
|  |  |  |  | **Lower** | **Upper** | **N** |
| Hydrocortisone 1% | Mometasone furoate | 0.02 | 0.987 | -1.95 | 1.99 | 55 |
| SNG100 | **Mometasone furoate** | **-1.76** | **0.040** | **-3.44** | **-0.08** |  |
| SNG100 | **Hydrocortisone 1%** | **-1.77** | **0.042** | **-3.48** | **-0.07** |  |
| Visit 1 | Visit 3 | 7.90 | <0.001 | 6.68 | 9.12 |  |
|  | Visit 4 | 9.04 | <0.001 | 7.76 | 10.31 |  |
|  | Visit 5 | 6.26 | <0.001 | 4.73 | 7.79 |  |

**Supplementary Table 6: Percent of participants achieving SCORAD change >75% (SCORAD75)**

| Visit # | Mometasone furoate | Hydrocortisone 1% | SNG100 | p value |
| --- | --- | --- | --- | --- |
| Visit 3  *No*  *Yes* | **n=18**  13 (72.2%)  5(27.8%) | **n=20**  15 (75.0%)  5 (25.0%) | **n=17**  11 (64.7%)  6 (35.3%) | NS |
| Visit 4  *No*  *Yes* | **n=18**  6 (33.3%)  12 (66.7%) | **n=20**  12 (60.0%)  8 (40.0%) | **n=17**  7 (41.2%)  10 (58.8%) | NS |
| Visit 5  *No*  *Yes* | **n=18**  11 (61.1%)  7 (38.9%) | **n=19**  17 (89.5%)  2 (10.5%) | **n=17**  11 (64.7%)  6 (35.3%) | NS |
