# Supplementary material for: SNG100, a novel topical treatment for moderate atopic dermatitis, in patients aged 6 years or older: A randomised, double‐blind, active‐controlled trial
Source: Skin Health Dis. 2023 Oct 14;3(6):e293. doi: 10.1002/ski2.293 (PMC10690700; doi:10.1002/ski2.293)
Supplement: Supplementary file 2 — Supporting Information S2 [file SKI2-3-e293-s001.pdf]

Subject Code Number:	Visit Date:
[ ][ ] — [ ][ ][ ]	D D M M Y Y

Select a suitable visit: ☐VISIT 3 TREATMENT ☐VISIT 4 TREATMENT

USABILITY QUESTIONNAIRE

		1	2	3	4	5
**1.**	**During the past week, Was the product easy to apply?**	1<br>Difficult	2	3	4	5<br>Easy
**2.**	**During the past week, did you find the product's texture pleasant?**	1<br>Unpleasant	2	3	4	5<br>Pleasant
3.	**During the past week, was the product quickly absorbed in your scalp?**	1<br>Not easily absorbed	2	3	4	5<br>Easily absorbed
4.	**During the past week, was the product sticky?**	1<br>Very sticky	2	3	4	5<br>Not sticky
5.	**During the past week, was the product greasy?**	1<br>Very greasy	2	3	4	5<br>Not greasy
6.	**During the past week, did the product leave a shiny look?**	1<br>Very shiny	2	3	4	5<br>Not shiny
**7.**	**During the past week, did you find the product convenient to use?**	1<br>Not convenient	2	3	4	5<br>Very convenient
**8.**	**During the past week, did the product stain your clothes?**	1<br>Stained badly	2	3	4	5<br>Did not stain
**9.**	**Do you think the product is suitable for applying over large areas?**	1<br>No	2	3	4	5<br>Yes

Comments: ____

____

____
